# Early continuous ultrafiltration in Chinese patients with congestive heart failure (EUC-CHF): study protocol for an open-label registry-based prospective clinical trial

**DOI:** 10.1186/s12872-019-1208-y

**Published:** 2019-11-07

**Authors:** Ying Yang, Chao Shen, Jiangting Lu, Fen Xu, Jinshan Tong, Jiangfen Jiang, Guosheng Fu

**Affiliations:** Department of Cardiology, Sir Run Run Shaw Hospital, College of Medicine, Zhejiang University, Zhejiang, Hangzhou China

**Keywords:** Early ultrafiltration, Heart failure, Fluid overload, Loop diuretics, Continuous renal replacement therapy, Inflammatory cytokines

## Abstract

**Background:**

Conventional pharmacologic therapies aim to reduce fluid overload in advanced heart failure (HF) represented by intravenous (IV) loop diuretics (LDs) have sometimes not so efficacious and been reported to have side effects such as unpredictable removal of water and sodium and electrolyte disturbance. It is not certain whether early ultrafiltration (UF) is effective than LDs in relieving edema. Given the weakness of evidence for early UF in patients with fluid overload, recommendations of UF in guidelines is considered as second-line therapy only for patients with refractory congestion, who failed to respond to LD-based strategies.

**Methods:**

The early continuous ultrafiltration in Chinese patients with congestive heart failure (EUC-CHF) trial is an open-label, registry-based, prospective study, recruiting patients with severe acute decompensated HF who are hospitalized for HF worsening due to overt fluid overload 24 h from hospital admission. Forty patients will be enrolled to two treatment groups (*n* = 20 for each group). The primary outcomes are the changes of weight loss and dyspnea severity score after treatment, as well as the occurrence of clinically overt major bleeding.

**Discussion:**

EUC-CHF trial was primarily designed to evaluate the efficacy and safety of early UF in patients with acute decompensated HF to reduce volume overload and improve clinical outcome. The trial aims to determine if early UF in acute HF is superior to IV LDs in clinical parameter improvement without adverse events and prevents rehospitalization up to 30 days. Also the trial is expected to establish a scoring system based on Chinese population to guide early UF treatment in appropriate patients. EUC-CHF is one of the first controlled trials tailored to determine the benefit of UF with 24 h from hospital admission.

**Trial registration:**

www.chictr.org.cn, ChiCTR1800019556. Registered on 18 November 2018.

## Background

Fluid overload and congestion are major causes for hospitalization in advanced heart failure (HF). Therapies aim to reduce fluid overload are the most important targets of treatment for acute decompensated HF patients. Intravenous (IV) loop diuretics (LDs) are widely accepted as the initial treatment for fluid removal, and are recommended in the guidelines for most of the patients with acute HF admitted due to fluid overload to improve symptoms. However, inappropriate use of high dose LDs may induce hypovolemia, hypotension, activation of the neurohumoral axis, worsening renal function and electrolyte disturbances. Also, up to 30% of the patients with decompensated HF (NYHA classification III to IV) present with LDs resistance [1, 2]. So far, LDs have never been systematically validated as safe and efficacious in reducing long-term mortality of HF due to lack of randomized controlled large-scale studies. The side effects and lack of long-term benefits restrain its use.

Growing evidence from clinical research indicate that ultrafiltration (UF) is effective in relieving pulmonary and peripheral edema for patients with severe fluid overload and LD resistance, as well as ameliorating haemodynamics and restoring diuretic responsiveness. Treatment with UF may represent a more rapid and physiologic method of fluid removal than LDs therapy. It is associated with a better effect of decongestion and haemodynamic variables improvement as well as longer clinical stabilization and a lower rehospitalization rate compared with LDs [3–5].

Currently, UF recommend by most of the guidelines are usually considered for patients with refractory congestion not responding to medical therapy. Previous research found although UF improve hemodynamics in acute decompensated HF refractory to standard medical therapy, it was also associated with high incidence of renal function damage and high in-hospital mortality [6]. However, other research found UF within 12 h of hospitalization in HF patients with volume overload and LD resistance before IV LDs effectively decreased length of stay and readmissions up to 3 months without change of renal function [7]. At present, most of the studies support UF should be considered as early treatment strategies (24 h after admission) in patients with severe HF instead of a “rescue therapy”. Early UF may prevent undesirable cardiorenal syndrome and sodium-volume overload before renal tubular damage induced by further neurohormonal activation and side effects caused by high dose LDs [8, 9].

Taken together, previous related studies showed UF is more effective in decongestion, haemodynamic variables and clinical outcome improvement, as well as restoring LD responsiveness compared with LDs. But most of the studies were based on the foreign population and did not define the exact time period from admission to UF. We do not have the related data for early UF in Chinese population. Furthermore, former study did not define how to screen out the patients recommended for early UF by specific scoring system. Our study aims to determine if early UF 24 h within admission in acute HF is superior to IV LDs in clinical parameter improvement without adverse events and prevents rehospitalization up to 30 days in Chinese population.

## Methods/design

### Aim

The early continuous ultrafiltration in Chinese patients with congestive heart failure (EUC-CHF) trial was designed to evaluate the efficacy and safety of early UF in patients with acute decompensated HF to reduce volume overload and improve clinical outcome. In addition, the study will determine risk factors help to screen out patients appropriate for early UF treatment.

### Study overview

The EUC-CHF trial is an open-label, registry-based, prospective, single center study. It will recruit patients with severe acute decompensated HF who are hospitalized for HF worsening and significant weight gain due to overt fluid overload 24 h from hospital admission. The determination that acute decompensated HF is the primary cause of hospitalization will be made by the site’s principal investigator or sub-investigator, based on the assessment of the clinical signs and symptoms as well as laboratory examinations that lead to the admission. Patient enrollment should be completed and study treatment initiated within 24 h from hospital admission. Patients will be enrolled 1:1 sequentially to receive standard medical therapy (control group) or early UF (early UF group). Up to 2 doses of IV LDs will be allowed before enrollment.

The diuretic protocol adopted by our study in the control group refers to the protocol of AVOID-HF and CARRESS-HF trial as well as the dose recommended by the guideline according to patients’ vital signs, status of hemoconcentration, and renal function (Table 1) [10, 11]. Patients of the early UF group will be treated with a single or repeated session of UF within 24 h from admission (according to local clinical practice and with availability of nursing surveillance during UF) until the relief of patient’s symptom. The UF protocol adopted by our study also refer to the protocol of AVOID-HF and CARRESS-HF trial (Table 2). 
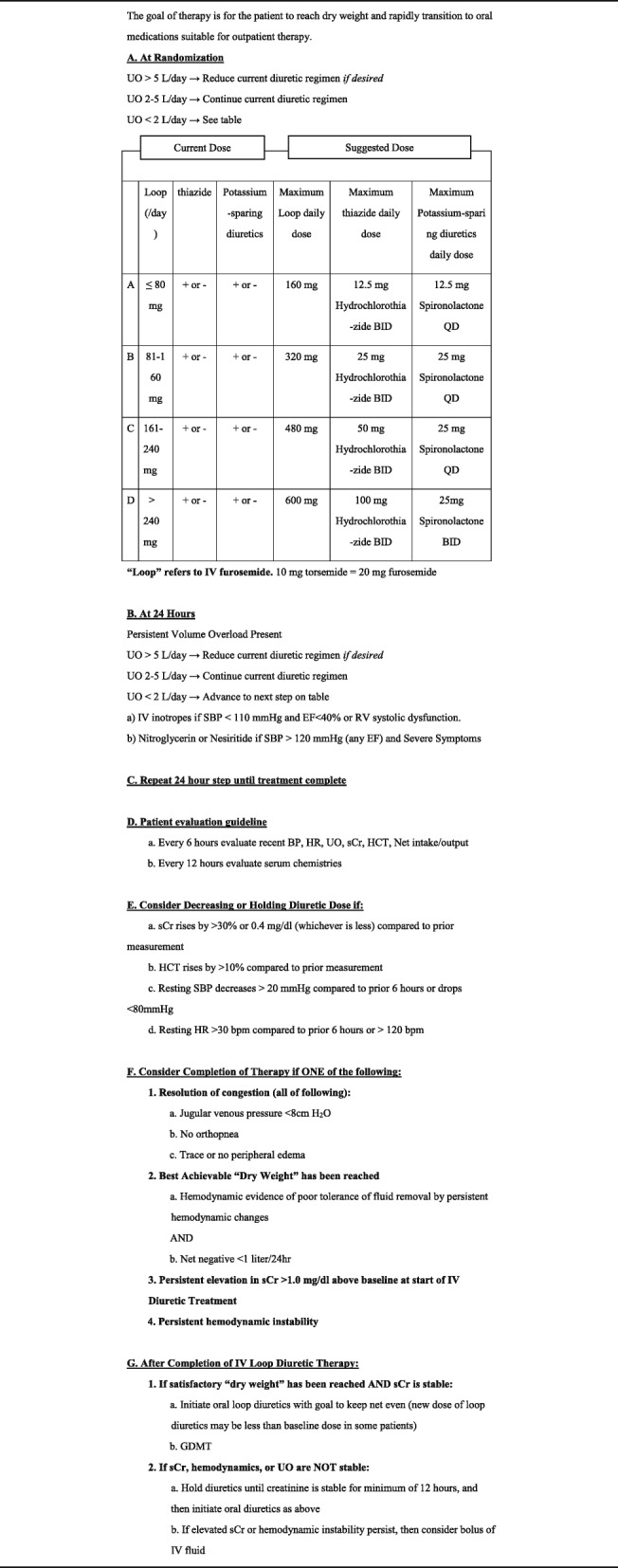

**Table 1:**
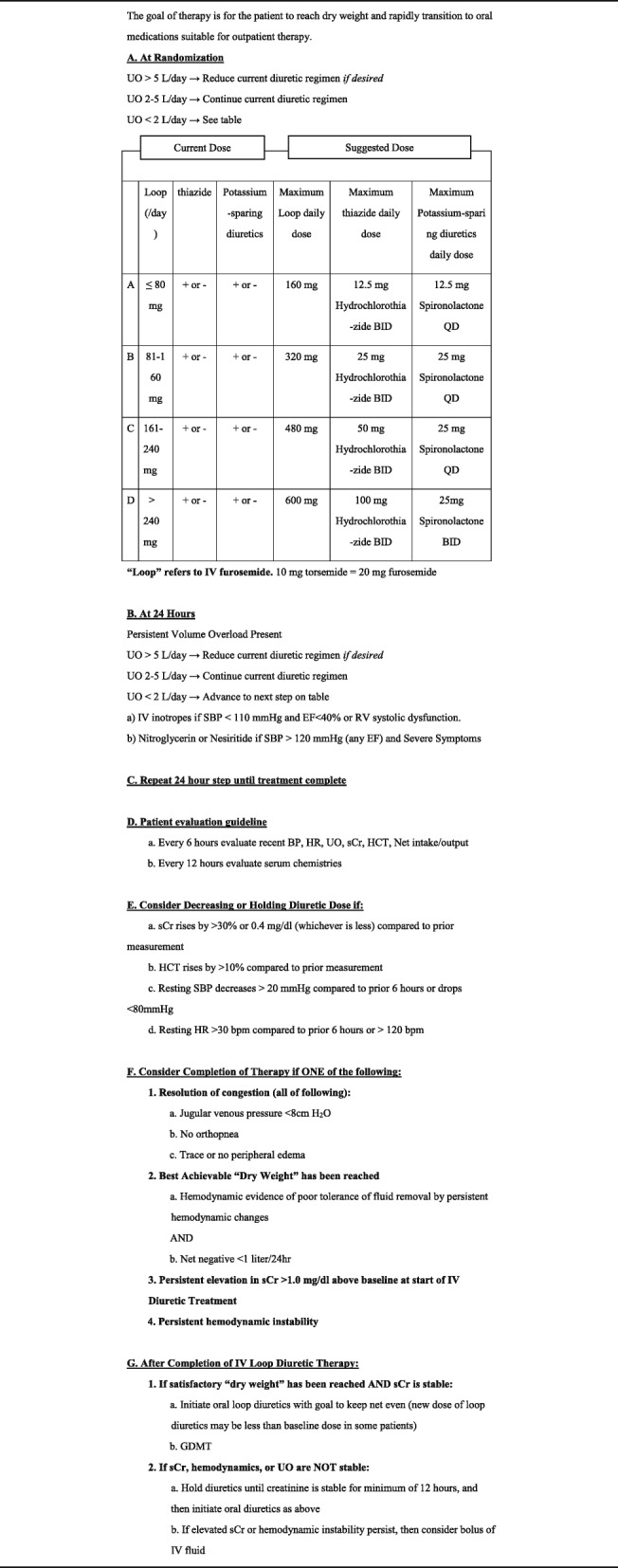
Treatment guidelines for the control group

Abbreviations: *BID* Twice daily, *BP* Blood Pressure, *BPM* Beats per Minute, *EF* Ejection Fraction, *GDMT* Guidelines Determined Medical Therapy, *HCT* Hematocrit, *HR* Heart Rate, *IV* Intravenous, *QD* Once daily, *RV* Right ventricle, *SBP* Systolic Blood Pressure, *sCr* Serum creatinine level, *UO* Urine Output

**Table 2:** Treatment guidelines for the early UF group

General Comments:	
**1.** Once an initial UF rate is chosen, avoid increasing the UF rate unless there are clear indications to do so. **2.** Because patients’ plasma refill rate usually declines as fluid is removed, it should be expected that UF rate will need to be decreased during the course of therapy. **3. a** IV inotropes if SBP < 110 mmHg and EF < 40% or RV systolic dysfunction. **b** Nitroglycerin or Nesiritide if SBP > 120 mmHg (any EF) and Severe Symptoms	
A. Choose Initial UF Rate:	
SBP < 100 mmHg: 150 cc/hr. SBP 100-120 mmHg: 200 cc/hr. SBP > 120 mmHg: 250 cc/hr	
B. Decrease starting UF rate by 50 cc/hr if any of the following are present:	
**a.** RV > LV systolic dysfunction **b.** sCr increase 0.3 mg/dl above recent baseline **c.** Baseline sCr > 2.0 mg/dl **d.** History of instability with diuresis or UF in the past	
C. Re-evaluate UF rate every 6 h:	
**1.** Evaluate recent BP, HR, UO, sCr, HCT, Net intake/output **2.** Consider decreasing UF rate by 50 cc/hr. and checking STAT sCr (unless sent in past 2 h) if: **a.** sCr rise > 15% or > 0.2 mg/dl (whichever is less) compared to prior measurement **b.** HCT rises by > 5% compared to prior measurement **c.** resting SBP decreases > 10 mmHg compared to prior 6 h, but remains > 80 mmHg **d.** UO drops > 50% compared to prior 6 h, but remains > 125 cc/6 h **e.** resting HR increases by > 20 bpm compared to prior 6 h, but remains < 120 bpm **3.** Strongly consider holding UF and checking STAT sCr if: **a.** sCr rise by > 30% or > 0.4 mg/dl (whichever is less) compared to prior measurement **b.** HCT rises by > 10% compared to prior measurement **c.** resting SBP decreases > 20 mmHg compared to prior 6 h or is < 80 mmHg **d.** UO < 125 cc/6 h **e.** resting HR increases by 30 bpm compared to prior 6 h or is > 120 bpm **4.** If UF held, re-evaluate after laboratory values are available: **a.** If hemodynamics is stable and sCr has plateaued, then consider re-starting UF at rate 50-100 cc/hr. less than previous rate **b.** If persistent volume overload is present, then consider: **i.** Adjusting doses of IV inotropes in patients with LVEF < 40% or RV systolic dysfunction **ii.** Weaning venodilators, especially in patients with HFpEF **iii.** Right heart catheterization	
D. Consider completion of UF therapy If ONE of the following occurs:	
**1.** Resolution of congestion (all of following): **a.** Jugular venous pressure < 8 cm H_2_O **b.** No orthopnea **c.** Trace or no peripheral edema **2.** Best Achievable “Dry Weight” has been reached **a.** Evidence of poor tolerance of fluid removal AND **b.** UF rate < 100 cc/hr. or net negative < 1 l/24 h **3.** Persistent elevation in sCr > 1.0 mg/dl above baseline at start of UF treatment **4.** Persistent hemodynamic instability	
E. After completion of UF Therapy:	
**1.** If satisfactory “dry weight” has been reached AND sCr is stable: **a.** Initiate oral loop diuretics with goal to keep net even (new dose of loop diuretics may be less than baseline dose in some patients) **b.** GDMT **2.** If sCr, hemodynamics, or UO are NOT stable: **a.** Hold diuretics until sCr is stable for minimum of 12 h, then: **i.** If “Dry Weight” /adequate decongestion has been reached then initiate oral diuretics as above **ii.** If “Dry Weight”/adequate decongestion has NOT been reached then initiate IV diuretics **b.** If elevated sCr or hemodynamic instability persist, then consider bolus of IV fluids	

Abbreviations: *BP* Blood Pressure, *BPM* Beats per Minute, *GDMT* Guidelines Directed Medical Therapy, *HR* Heart Rate, *IV* Intravenous, *LV* Left Ventricle, *RV* Right ventricle, *SBP* Systolic Blood Pressure, *sCr* Serum creatinine level, *UF* Ultrafiltration, *UO* Urine Output

During the process of UF, hematocrit will be monitored to reduce the risk of hypovolemia-induced acute kidney injury associated with excessive dehydration. In both group, protocol of inotropics and vasodilators use for each group are listed in Table 1 and 2. Other additional medical therapy will be left to the discretion of the cardiologist responsible for the patient. IV LDs started before enrollment will be discontinued during the process of UF. During the follow-up period, physicians can modify LDs dose according to patients’ situation according to the diuretic protocol.

UF will be performed with the FQ-16 HF UF dewatering device (HeartcareMedical Technology Co., Ltd., Beijing, China). Patients in the UF group will receive heparin to achieve an activated partial thromboplastin time (APTT) 1.5 to 2.5 times normal (or ACT180–220 s) or low molecular heparin according to routine clinical practice without monitoring APTT. The vascular access will be obtained by placing a double-lumen catheter (≥ 8 Fr) in a major venous vessel. The UF rate could be changed during the procedure according to the clinical situation (see in Table 2).

**Fig. 1:**
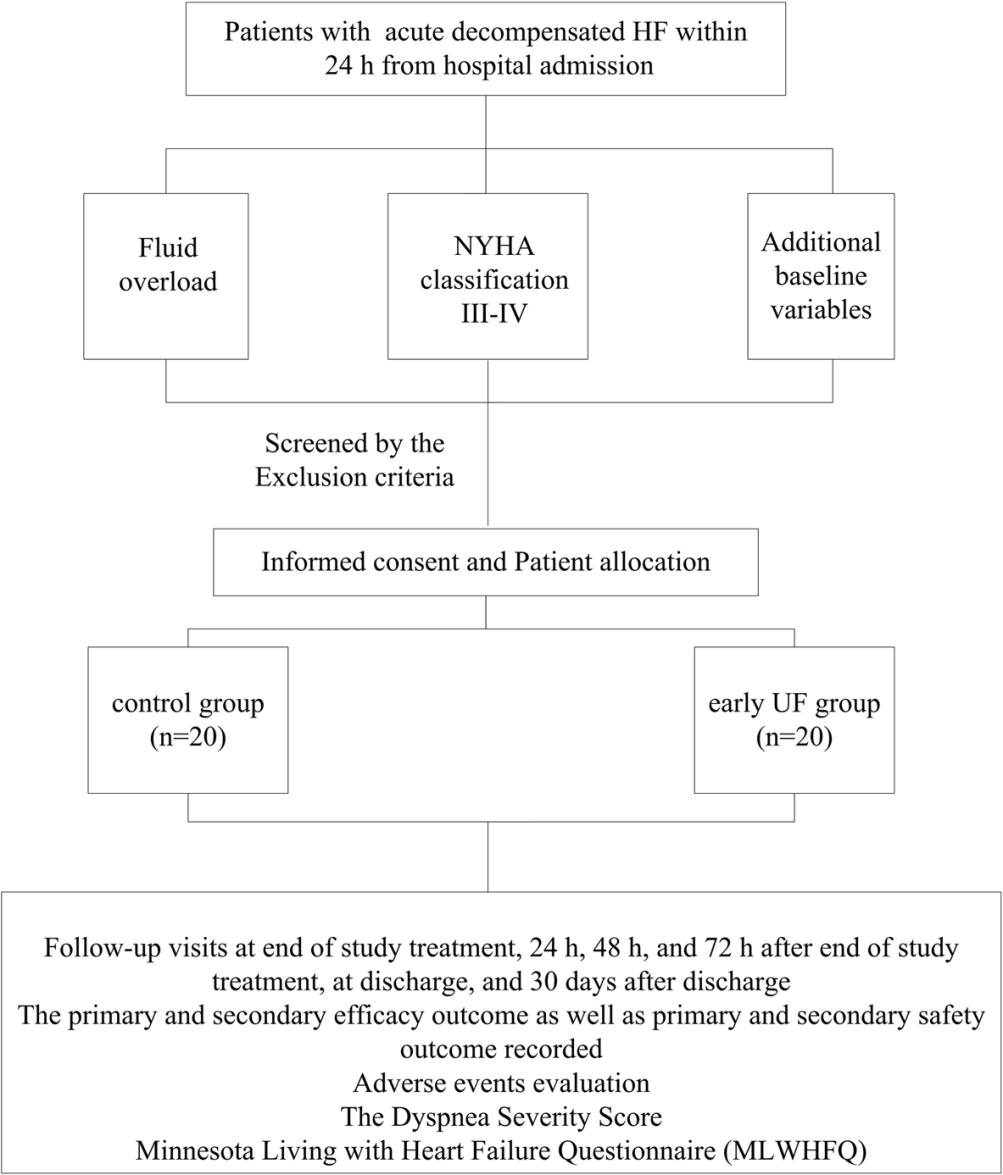
Study design. The control group will be treated with intravenous loop diuretics by experienced HF cardiologists according to guideline recommendations. Patients of the early UF group will receive a single or repeated session of UF started within 24 h from admission until the relief of patient’s symptom. HF, heart failure; UF, ultrafiltration

Key inclusion and exclusion criteria are shown in Table 3. The study design and schedule of follow-up visit are summarized in Fig. 1. EUC-CHF is registered at www.chictr.org.cn (ChiCTR1800019556). The trial started recruitment in December 2018 and enrolment of the first participant to the trial is on 14 December 2018.

**Table 3:** Key inclusion and exclusion criteria of the study

Inclusion criteria	1. Patient must be more than 18 years old. 2. Acute decompensated HF within 24 h of hospital admission. 3. Fluid overload, defined as edema of the lower extremities and at least 1 of the following: 1) elevated jugular venous pressure > 10 cm H_2_O, 2) pulmonary rales, paroxysmal nocturnal dyspnea or orthopnea, 3) pulmonary edema or pleural effusion on chest X-ray, 4) enlarged liver or ascites, 5) pulmonary wedge or left ventricular end diastolic pressure ≥ 20 mmHg, or 6) rapid increase of body weight ≥ 2 kg. 4. NYHA classification III-IV. 5. Patient is informed and has signed informed consent. Patient is willing to attend an outpatient follow-up.
Exclusion criteria	1. Acute coronary syndrome. 2. Severe mitral or aortic stenosis. 3. Severe renal insufficiency (serum creatinine ≥3.0 mg/dl or eGFR ≤ 30 mL/min). 4. Systolic blood pressure ≤ 90 mmHg or depending on high dose of vasoactive drug to maintain systolic blood pressure ≥ 90 mmHg. 5. Hematocrit > 45%. 6. Unattainable venous access. 7. Comorbidities expected to prolong hospitalization. 8. Contraindications to anticoagulation. 9. Serious bleeding in the last 6 months (this includes, but is not limited to, prior intracranial hemorrhage, active peptic ulcer disease, active internal bleeding, bleeding at a noncompressible site). 10. Hematuria within 30 days before randomization. 11. Patients at high risk of bleeding (this includes, but is not limited to, clinically significant thrombocytopenia or anemia, platelet count < 90,000/μL at screening or pre-randomization, anemia of unknown cause with a hemoglobin level < 10 g/dL at screening or pre-randomization, coagulation disorders, recent stroke within past 30 days, documented hemorrhagic tendencies, or blood dyscrasias). 12. Uncontrolled hypertension (> 180/110 mmHg). 13. Severe infection. 14. Use of iodinated radiocontrast material. 15. Participation in another controlled clinical trial, either within the past 2 months or still ongoing. 16. Women who are pregnant, breast-feeding, or of child-bearing potential. 17. Patient who is mentally ill.

The trial aims to recruit 40 patients (20 patients for each group) over an estimated 24-month period. After baseline visit, follow-up visit will occur at the end of study treatment, 24 h, 48 h, and 72 h after the end of study treatment, at discharge, and 30 days after discharge. At the baseline visit, informed consent will be taken, and additional baseline variables including gender, age, height, weight, occupation, race, physical activity, complete medical history (including history of smoking and drinking, past and present medical history, concomitant disease), vital signs, NYHA classification, dyspnea severity score, score by Minnesota Living with Heart Failure Questionnaire (MLWHFQ) functional capacity, physical examination including distention of jugular vein, edema of lower extremity and congestion of other organs, laboratory test, electrocardiogram, chest X-ray, echocardiographic data and concomitant drug used up to 3 months before enrollment will be recorded.

Questionnaires (dyspnea severity score and score by MLWHFQ functional capacity) at follow-up visits will also be collected by trained clinicians. This will aid in our assessment of patients’ cardiac function and other safety data.

During follow-up, we will record all the primary and secondary end points and adverse events. All adverse events will be evaluated according to severity, causality, expectedness, and relationship to study treatment. Subjects who develop complications that, in the judgment of principal investigator or sub-investigator, are not suitable to continue the trial will be screened out from the study. Participants will continue with follow-up and may restart study protocol if the clinical situation resolves. Investigators are responsible for the decision to resume the study. If treatment according to the study protocol is clinically insufficient, the study will be suspended and effective treatment should be applied according to the current clinical practice.

Blinding cannot be realized because of different decongestion methods used in the 2 groups. A researcher who is unaware of the treatment group will adjudicate all endpoint events and another researcher who is also blinded to the patient allocation is assigned for the statistics task.

### Study outcomes

The primary end points of EUC-CHF are the changes of weight loss and dyspnea severity score at every scheduled visit which are considered as the part of a single composite endpoint, as well as the occurrence of clinically major bleeding defined by TIMI or GUSTO criteria [12].

Secondary outcomes include: net fluid loss, length of stay in CCU or ICU, change of score by MLWHFQ functional capacity, change of serum and ultrafiltrate concentrations of endotoxin, ET-1, TNF-a, IL-6 and IL-10, changes of cardiac function (the NYHA classification), changes in N-terminal proB-type natriuretic peptide (NT-proBNP) level, changes of LDs dose, HF rehospitalizations for congestive HF in 30 days after discharge, cardiovascular death and all-cause death in 30 days after discharge, change of blood pressure, changes in hepatic and renal function (serum creatinine concentration, estimated glomerular filtration rate [eGFR]), serum electrolytes, hemoglobin and incidence of acute coronary syndrome at scheduled visits.

Weights will be obtained at every scheduled visit using the same balance with the patient shoeless in a hospital gown after emptying the bladder. Weight loss is the difference between weight at baseline and the weight recorded at subsequent visits.

The dyspnea severity score by a proposal to standardize dyspnea measurement in clinical trials of acute heart failure syndromes by International Working Group on acute heart failure of European Society of Cardiology is used for the assessment of dyspnea [13].

Total fluid intake and output (ultrafiltrate and urine) measured at every scheduled visit after enrollment will be used to calculate net fluid losses.

Glomerular filtration rate will be calculated according to the abbreviated Modification of Diet in Renal Disease (MDRD) equation [14].

Acute coronary syndrome includes unstable angina, ST segment elevated myocardial infarction and non-ST segment elevated myocardial infarction. Myocardial infarction is defined according to the fourth universal definition of myocardial infarction [15]. Death will be considered cardiac according to the 2014 ACC/AHA key data elements and definitions for cardiovascular endpoint events in clinical trials [16].

We also will collect all the urine output after patients are enrolled to receive standard medical therapy or early UF until 72 h after the end of study treatment. Then we will measure the average urine electrolytes for every enrolled patient.

### Statistical analysis

We will compare estimates of patients who receive UF therapy with those who receive conventional pharmacologic therapy. Concomitant medication and other baseline characteristics will be utilized in subgroup analysis.

Continuous variables will be summarized using descriptive statistics. Qualitative variables will be summarized by counts and percentages.

We will compare baseline characteristics. T-test or sign rank test will be used after testing for normality. Chi-square or Fisher’s exact test will be performed for categorical variables.

The primary efficacy endpoint (change of weight loss and dyspnea severity score) will be analyzed by Kaplan-Meier method, and comparisons between two groups will be performed using a log-rank test. Hazard ratio with 95% CI will be derived by the Cox proportional hazards model. Subgroup analyses of the efficacy endpoint are performed for the evaluation of impact of major clinical features on the improvement of primary efficacy endpoints by early UF therapy. Specific information is as follows:

1. The ability to predict the therapeutic effect of UF by patients’ individual characteristics as well as systolic blood pressure, heart rate, severity of edema, renal and hepatic function, NYHA classification, left ventricular ejection fraction, chamber diameter, concomitant disease and medication;
2. The influence of different patients’ individual characteristics including gender, age, height and weight on the effect of UF for fluid removal and alleviation of dyspnea;
3. The influence of baseline systolic blood pressure and heart rate on the effect of UF for fluid removal and alleviation of dyspnea;
4. The influence of baseline severity of edema (i.e., edema of lower extremity and congestion of other organs) on the effect of UF for fluid removal and alleviation of dyspnea;
5. The influence of baseline renal and hepatic function on the effect of UF for fluid removal and alleviation of dyspnea;
6. The influence of different baseline cardiac function (including NYHA classification and left ventricular ejection fraction) on the effect of UF for fluid removal and alleviation of dyspnea;
7. The influence of different baseline chamber diameter on the effect of UF for fluid removal and alleviation of dyspnea;
8. The influence of different baseline concomitant disease on the effect of UF for fluid removal and alleviation of dyspnea;
9. The influence of different concomitant medication on the effect of UF for fluid removal and alleviation of dyspnea;

Subgroup analysis of primary efficacy endpoint will be estimated using a stratified Cox model. The subgroup analysis will be tested by chi-square or Fisher’s exact tests.

The primary safety endpoints and secondary endpoints will be presented by summary statistics, as number of patients, event counts and percentage, variable counts and percentage.

All comparisons will report *p* values on the basis of 2-sided tests, and a p value ≤ 0.05 will be considered statistically significant. All statistical analyses will be performed using SPSS Statistics version 22 software.

## Discussion

In the EUC-CHF study, we investigate whether early UF (with 24 h after admission) in patients hospitalized for HF worsening and significant fluid overload could help to relieve volume overload and symptom, as well as decrease short-term recurrence of acute HF and rehospitalization.

So far most of the previous researches focused on the benefits of UF in population with LD resistance. A retrospective cohort study from Asian population found UF in patients with decompensated HF and LD resistance achieved lower urine output, greater fluid loss, greater weight loss and a shorter duration of hospitalization than the standard care group. At 90 days, the UF group had fewer emergency department visits and rehospitalizations for HF [17]. In patients with acute decompensated HF due to fluid overload who whether or not have LD resistance, it is unclear about the appropriate time required to start UF. Previous randomized controlled trials (RAPID-CHF, UNLOAD, CUORE and AVOID-HF trials) compared UF to usual care with IV LDs in hospitalized patients with congestion and decompensated HF. Some of the enrolled patients received UF within the first 24 h of admission. The results from these trials all indicated that early UF safely and effectively reduced congestion, decreased length of stay, HF rehospitalizations, and cardiovascular events in acute HF patients. Clinical benefits of early UF in some of these trials last for 3 months to 1 year [4, 8, 18, 19]. Renal function changes were similar between UF and IV LDs in all of these trials. Two studies in acute renal failure after cardiac surgery demonstrated that early recognition of acute renal failure and initiating UF at early-stage are of vital importance. Early UF reduced in-hospital mortality in postcardiac surgery patients with acute renal failure [20, 21]. However, when UF was initiated late during hospitalization, particularly in patients who had already developed acute kidney injury, as in the CARRESS-HF trial, UF may lose its advantage over pharmacologic therapy. This indicated that UF should be considered as an early strategy rather than a rescue treatment. UF stated late when patients developed hemodynamic deterioration may be of less benefit and associated with a higher rate of adverse events such as renal dysfunction [22]. So we will enroll patients with acute decompensated HF due to fluid overload within 24 h after admission.

Currently, clinical UF use differs greatly among different centers, countries and physicians due to widely accepted consensus or guidelines. Bagshaw SM et al. proposed an algorithm aimed to evaluate when to initiate RRT. The algorithm included some patient-related factors, which may influence the time point of initiating RRT [23]. Equally, a clinical scoring system that is based on the patient’s clinical and laboratory information on admission as well as other potential risk factors obtained from present available trials may be useful for screening out patients suitable for UF. Using this system probably contribute to avoid unnecessary risks of UF. Amir Kazory et al. suggested a number of potential factors such as the primary renal structural disease, age, ejection fraction, blood pressure, serum sodium level, BNP level, presence of right-sided HF, history of LD resistance in previous hospitalizations, and progression of congestion despite high-dose LDs may be used as predictors for the responders from UF [24]. So our study will record baseline characteristics for subgroup analysis. We expect to find the risk factors associated with the non-responsiveness to UF. Also, we try to seek out certain patient-based clinical and laboratory parameters that could help to screen out patients appropriate for early UF.

Although experts and previous evidence from several randomized trials support the view that early UF resulted in more body fluid depletion, the timing of UF in acute HF patients in most trials are not considered as a risk factor [25]. It is not certain when and for which population is suitable to start UF. Further studies are needed to determine the clinical significance of timing for UF. So far there is no such research in China. In the present study, we will analyze the efficacy of early UF (within 24 h after admission) versus conventional pharmacologic therapy in Chinese patients with severe decompensated HF who are hospitalized for HF worsening and overt fluid overload. Thereafter, we hope to establish a scoring system based on Chinese population with acute decompensated HF to screen out appropriate candidates for early UF with the purpose of achieving better volume depletion and clinical outcome improvement. The scoring system may consist of the timing of UF as well as certain patient-based clinical and laboratory parameters.

Additionally, patients with edema due to acute HF had higher concentrations of endotoxin and cytokines [26–28]. Bacterial or endotoxin might translocate from the bowel into the blood stream in HF. Endotoxin may trigger immune activation in patients developed edema due to HF [27]. Continuous renal replacement therapy (CRRT) can reduce circulating cytokines and inflammatory mediators that result in ameliorating the central and peripheral manifestations of CHF [29]. CRRT after cardiac operations in children and adult could not only remove the fluid overload but also eliminate inflammatory mediators. It has been shown to improve hemodynamics, hemostatic dysfunction, pulmonary functions, and clinical outcome such as early morbidity [30–32]. CRRT is also effective in reducing BNP and circulating cytokines levels including IL-8 and monocyte chemoattractant protein-1 (MCP-1), as well as help patients regaining responsiveness to LDs in in a very small advanced HF population (5 patients) [33].

Although theoretically UF specific filter membrane could only penetrate small molecules such as sodium and water, there is no previous evidence from clinical studies on whether UF could filter out inflammatory mediators. It is not exactly certain whether UF is able to remove inflammatory mediators. Previous research found that high-volume continuous veno-venous hemofiltration combined with periodic filter change, different UF rates, different membrane pore sizes, different types of membrane, and different species were all related to the efficacy of removing inflammatory mediators by hemofiltration [34–36]. The capacity of filtering out mediators by UF specific filter membrane is influenced by many factors, such as pore size, screening coefficient of the membrane, molecular charge and molecular weight of the mediators. It is difficult to determine whether they can be filtered out only by the molecular weight of the mediators alone. Therefore, we will measure changes of serum and ultrafiltrate concentrations of inflammatory mediators including endotoxin, endothelin-1 (ET-1), tumor necrosis factor- (TNF-a), interleukin-6 (IL-6) and IL-10 in our study. We try to find out whether UF could relieve both fluid overload and other clinical manifestations such as HF related multiple organ dysfunction syndrome (MODS) in acute HF by filtering out certain inflammation mediators. Our study will provide further data regarding the ability of UF to filter out inflammation mediators. To our knowledge, this is the first trial exploring the effects of UF on circulating inflammatory mediators.

The present study is mainly limited by its open-label design and patient selection bias cannot be excluded, which will make the interpretation of the results somewhat limited. Also, the follow-up period is relatively short in our trial. Postpone the last clinical evaluation at 90 days and at an even later time point may provide more information of the advantages or disadvantages of UF.

In conclusions, this study evaluates the effectiveness and safety of selecting patients who are suitable and could benefit from early UF by certain risk factors to guide early UF treatment. Our research will provide clinical evidence for early UF in Chinese patients with acute decompensated HF and may contribute to volume overload reduction and clinical outcome improvement in this population. It also can help to prevent delay by clinicians to implement UF in the current domestic clinical practice of low rate of UF.

## Data Availability

Not applicable, the data has not been collected yet.

## List of Abbreviations

APTT: Activated partial thromboplastin time
CRRT: Continuous renal replacement therapy
eGFR: Estimated glomerular filtration rate
ET-1: Endothelin-1
HF: Heart failure
IL-6: interleukin-6
IV: Intravenous
LDs: Loop diuretics
MCP-1: Monocyte chemoattractant protein-1
MDRD: Modification of diet in renal disease
MLWHFQ: Minnesota living with heart failure questionnaire
MODS: Multiple organ dysfunction syndrome
NT-proBNP: N-terminal pro B-type natriuretic peptide
RRT: Renal replacement therapy
TNF-a: Tumor necrosis factor- a
UF: Ultrafiltration
